# Research on the cascading mechanism of “urban built environment-air pollution-respiratory diseases”: a case of Wuhan city

**DOI:** 10.3389/fpubh.2024.1333077

**Published:** 2024-03-22

**Authors:** Zhiqi Zhang, Yue Ding, Ruifeng Guo, Qi Wang, Yanfei Jia

**Affiliations:** ^1^School of Architecture and Urban Planning, Huazhong University of Science and Technology, Wuhan, China; ^2^Hubei Engineering and Technology Research Center of Urbanization, Wuhan, China; ^3^Department of Geriatrics, Tongji Hospital, Tongji Medical College, Huazhong University of Science and Technology, Wuhan, China

**Keywords:** air pollution, respiratory diseases, distributed lag non-linear models, multiscale geographically weighted regression model, Wuhan city

## Abstract

**Background:**

Most existing studies have only investigated the direct effects of the built environment on respiratory diseases. However, there is mounting evidence that the built environment of cities has an indirect influence on public health via influencing air pollution. Exploring the “urban built environment-air pollution-respiratory diseases” cascade mechanism is important for creating a healthy respiratory environment, which is the aim of this study.

**Methods:**

The study gathered clinical data from 2015 to 2017 on patients with respiratory diseases from Tongji Hospital in Wuhan. Additionally, daily air pollution levels (sulfur dioxide (SO_2_), nitrogen dioxide (NO_2_), particulate matter (PM_2.5_, PM_10_), and ozone (O_3_)), meteorological data (average temperature and relative humidity), and data on urban built environment were gathered. We used Spearman correlation to investigate the connection between air pollution and meteorological variables; distributed lag non-linear model (DLNM) was used to investigate the short-term relationships between respiratory diseases, air pollutants, and meteorological factors; the impacts of spatial heterogeneity in the built environment on air pollution were examined using the multiscale geographically weighted regression model (MGWR).

**Results:**

During the study period, the mean level of respiratory diseases (average age 54) was 15.97 persons per day, of which 9.519 for males (average age 57) and 6.451 for females (average age 48); the 24 h mean levels of PM_10_, PM_2.5_, NO_2_, SO_2_ and O_3_ were 78.056 μg/m^3^, 71.962 μg/m^3^, 54.468 μg/m^3^, 12.898 μg/m^3^, and 46.904 μg/m^3^, respectively; highest association was investigated between PM_10_ and SO_2_ (*r* = 0.762, *p* < 0.01), followed by NO_2_ and PM_2.5_ (*r* = 0.73, *p* < 0.01), and PM_10_ and PM_2.5_ (*r* = 0.704, *p* < 0.01). We observed a significant lag effect of NO_2_ on respiratory diseases, for lag 0 day and lag 1 day, a 10 μg/m^3^ increase in NO_2_ concentration corresponded to 1.009% (95% CI: 1.001, 1.017%) and 1.005% (95% CI: 1.001, 1.011%) increase of respiratory diseases. The spatial distribution of NO_2_ was significantly influenced by high-density urban development (population density, building density, number of shopping service facilities, and construction land, the bandwidth of these four factors are 43), while green space and parks can effectively reduce air pollution (*R*^2^ = 0.649).

**Conclusion:**

Previous studies have focused on the effects of air pollution on respiratory diseases and the effects of built environment on air pollution, while this study combines these three aspects and explores the relationship between them. Furthermore, the theory of the “built environment-air pollution-respiratory diseases” cascading mechanism is practically investigated and broken down into specific experimental steps, which has not been found in previous studies. Additionally, we observed a lag effect of NO_2_ on respiratory diseases and spatial heterogeneity of built environment in the distribution of NO_2_.

## 1 Introduction

The impact of air pollution on public health has been repeatedly studied, especially on respiratory diseases (1–5). Many studies have shown that air pollutants such as sulfur dioxide (SO_2_), nitrogen dioxide (NO_2_), particulate matter (PM_2.5_, PM_10_), and ozone (O_3_) are positively associated with mortality and morbidity from respiratory diseases (6–8). Air pollution poses a health risk to approximately 87% of the world’s population. In China, astounding economic and social development has led to rapid urbanization, accompanying serious air pollution and health problems (9). Previous studies on air pollution and respiratory diseases were mainly focused on European and American advanced countries, although in recent years, there has been an increase in related studies in China, but mainly in industrial cities and other relatively polluted cities, such as Beijing (10, 11), Jinan (12), Lanzhou (13, 14), Chongqing (15, 16), with limited research on air pollution and respiratory diseases in other cities, especially in central China. Wuhan, as a typical big city in central China, has a long history of serious air pollution and the resulting respiratory diseases are very common. Therefore, conducting a study on air pollution and respiratory diseases in Wuhan has important demonstrative significance for cities in central China.

Although respiratory diseases are frequently studied in public health fields about air pollution. Still, other research points to a possible connection between respiratory diseases and the built environment of cities. A study from Shanghai, China found that land use mix, building width-height ratio, frontal area density, and arterial road density were significantly correlated to the mortality of chronic obstructive pulmonary disease (COPD) in high-density urban areas (17). Another study from Indonesia found that housing quality, such as housing crowdedness and ventilation, and neighborhood conditions like neighborhood transportation modes and air pollution levels were significantly correlated to respiratory infectious diseases (18). The mechanisms of air pollution on respiratory diseases are familiar to the discipline of public health, yet the mechanisms of built environment elements on respiratory diseases are currently unknown.

Analyzed from another perspective, numerous studies in the field of urban environment have shown a close relationship between the built environment and air pollution. Land use, building density, road network density, and public green space have significant effects on the distribution and dispersion of air pollutants (19–23).

Is it possible, then, that air pollution is the means by which these features of the built environment impact respiratory diseases? In other words, air pollution becomes a mediating component in this process. Thankfully, research backs this up. This study demonstrates how variations in the built environment of cities may affect the concentrations of air pollutants, which can lead to a range of respiratory health issues (24). Regretfully, few researchers have carried out these kinds of investigations at the practical level; instead, they are primarily theoretical. Therefore, in order to examine the process by which elements of the built environment affect respiratory disorders, specialized research at the practical level must be conducted.

In addition, in the selection of the study scale, studies related to the urban built environment and respiratory health have mainly focused on urban areas in China. However, studies have shown that air pollutant concentrations vary in different urban spaces (25–27). Since the effects of built environment factors on air pollutant concentrations tend to vary by location (28), the effects of air pollution on respiratory disease within a region should be based on its sphere (29). Therefore, it is necessary to target the entire city to guide the formulation of public health policies that reflect regional differentiation.

To synthesize the above analysis, this study takes Wuhan as an example to analyze the influence of built environment elements on respiratory diseases from the practical level. The whole study is divided into four main steps: firstly, through the analysis of existing studies, we put forward the cascading mechanism of “urban built environment-air pollution-respiratory diseases,” and divide it into three analysis steps. In the second step, the direct impact of the built environment on respiratory diseases was analyzed. In the third step, the Distributional lag non-linear model (DLNM) is used to analyze the association between air pollutants, meteorological factors and respiratory diseases and the lag effect. In the fourth step, the spatial heterogeneity effect of built environment factors on the distribution of air pollutants was analyzed using the multiscale geographically weighted regression (MGWR) model. Since the direct effects of the built environment on respiratory diseases have been observed in our previous study (30), this study focuses the experiment on the third and fourth steps.

## 2 Materials and methods

### 2.1 Study area

Wuhan is the capital of Hubei Province and one of the largest cities in central China. The city spans over an area of 8,569 square kilometers, with a built-up area of 885 square kilometers, and comprises 13 administrative districts. The city has a permanent population of 13.65 million, and its rapid urbanization over the years has led to a range of urban problems while promoting economic growth. However, this has also led to environmental issues such as high levels of industrial pollution, car exhaust emissions, and waste incineration, which have worsened air pollution and severely impacted residents’ quality of life, particularly their respiratory health. To address these issues, the government has prioritized air pollution control and aimed to promote the development of a healthier and more sustainable urban environment. This study aims to investigate the relationship between the built environment, air pollution, and respiratory diseases in Wuhan, and contribute to urban planning efforts aimed at creating a better, healthier future.

### 2.2 Data collection

#### 2.2.1 Hospitalized patient data

This study obtained hospitalization data from January 1, 2015 to December 31, 2017, of 20,071 patients with respiratory diseases in Tongji Hospital in Wuhan. The data includes information such as a residential address, admission date, discharge date, gender, age, and diagnosis description. A total of 5,941 patient cases were analyzed, excluding those who did not reside in the local area. In our study, patient data were collected at Tongji Hospital’s clinical record, while written informed consent was obtained from all participants. The whole study was conducted in compliance with the Declaration of Helsinki, the study was approved by the Human Assurance Committee of Tongji Hospital (IRB: TJ-IRB20210942). We also took appropriate measures to protect participant privacy and data security during data analysis, all personally identifiable information was anonymized.

Causes of respiratory disease were classified according to codes in the *International Classification of Diseases*, *10th Revision* (ICD-10) and *11th Revision* (ICD-11) codes. We used daily hospitalization counts of acute upper respiratory infections of multiple and unspecified sites (ICD-11 codes CA07 and ICD-10 codes J06), certain lower respiratory tract diseases (ICD-11 codes CA20 to CA24 and ICD-10 codes J20 to J22), acute bronchitis (ICD-11 codes CA42).

#### 2.2.2 Air quality and meteorological data

**Figure 1:**
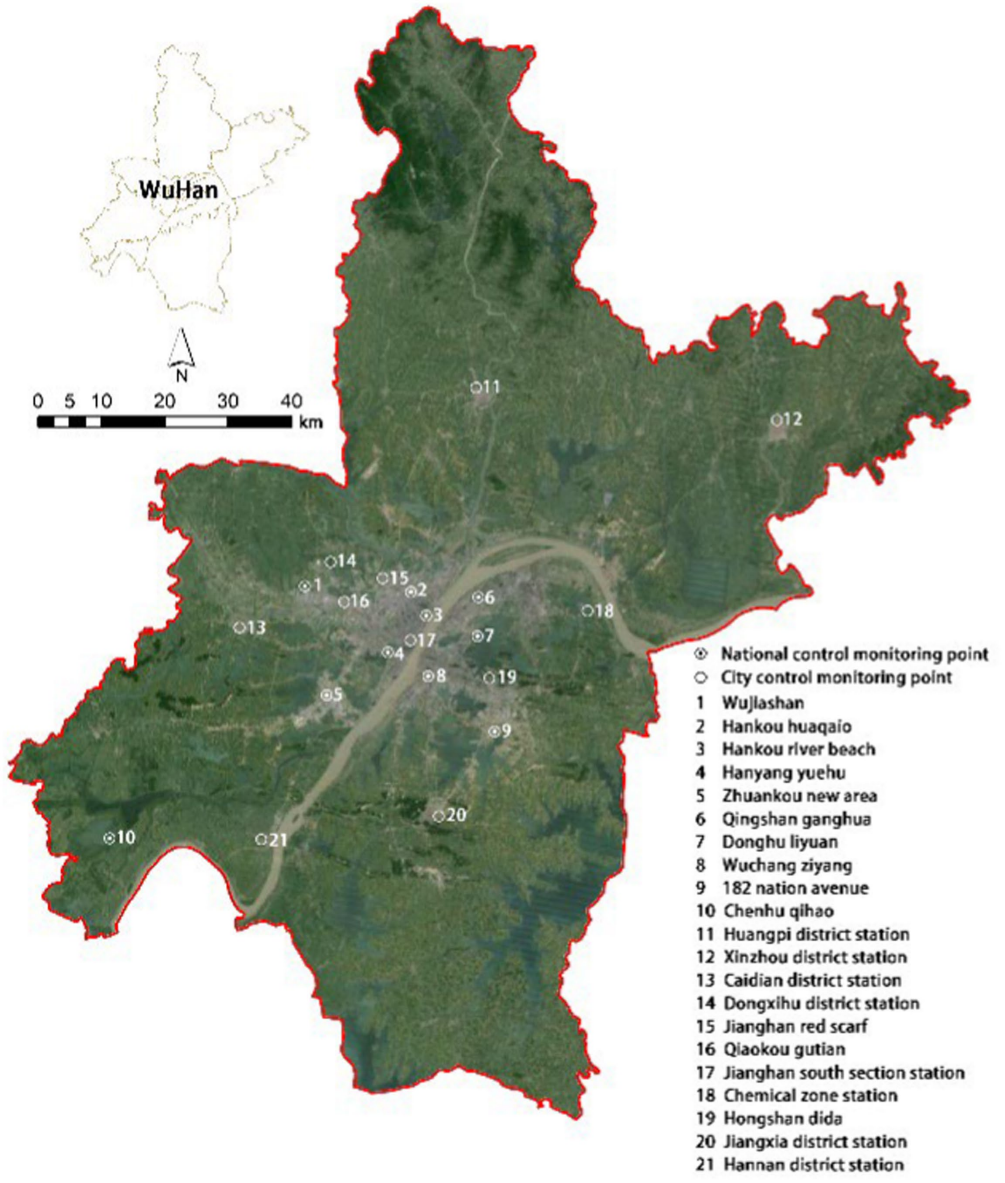
Distribution of 21 air quality monitoring stations in Wuhan.

Daily air quality data, which are calculated in terms of concentration on a daily basis, were obtained from 10 Chinese national meteorological monitoring stations in Wuhan and 11 of Wuhan’s own municipal meteorological monitoring stations (Figure 1), from January 1, 2015 to December 31, 2017. Daily air quality data detected at these stations are integrated into the official website of the Wuhan Bureau of Ecology and Environment^1^, from which we obtain air pollutants data (PM_2.5_, PM_10_, NO_2_, SO_2_, O_3_). The spatial distribution of air pollutants in Wuhan was obtained using the Kriging interpolation method, and the concentration of various air pollutants in each street was calculated. Daily meteorological data (average temperature and relative humidity) for the same period were obtained from the official website of the Wuhan Bureau of Ecology and Environment.

#### 2.2.3 Built environment data

The impact of the built environment on respiratory health is scrutinized by examining the following factors: residential density, green landscapes, and road traffic. The data used in this study consists of population data, building data, land use data, and road network data. Population data, sourced from the Wuhan Statistics Bureau’s “Wuhan Statistical Yearbook” website, was used to calculate population density at the street level. Building data, road network data, and traffic facility point data were obtained from the Baidu Map platform. The study predominantly used primary and secondary roads in the analysis. We collected land use data for Wuhan city from the 2017 National Land Use Cover dataset published by Prof. Gong Peng of Tsinghua University (31).

### 2.3 Data analysis

#### 2.3.1 Spearman’s correlation analysis

This study investigates the correlation between air pollutants and meteorological factors. We analyzed five air pollutants (sulfur dioxide (SO_2_), nitrogen dioxide (NO_2_), particulate matter (PM_2.5_, PM_10_), and ozone (O_3_)) and their daily average concentrations (measured in μg/m^3^). We also analyzed two meteorological factors, average temperature and relative humidity (RH) in this study. Spearman is calculated by the following Eq. 1:


(1) $$r_{s}=1-\frac{6\sum d_{i}^{2}}{n\left(n^{2}-1\right)}$$

In this formula, $r_{s}$ represents the Spearman correlation coefficient between two random variables, n is the number of studied days (number of studied days for air pollutant or meteorological factor data), and $d_{i}$ represents the difference in ranks corresponding to the two variables. The value of $r_{s}$ ranges from −1 to 1. When the correlation coefficient is 1, it indicates that the two variables are perfectly positively correlated; when the correlation coefficient is 0, it indicates that there is no linear correlation between the two variables; when the correlation coefficient is −1, it indicates that the two variables are perfectly negatively correlated.

#### 2.3.2 Analysis of distributed lag non-linear models

To address the non-linear relationship between air pollutants, meteorological factors, and respiratory health, this study applied a Poisson regression combined with the distributed lag non-linear model (DLNM), which was analyzed using R 4.1.0 (32–34). The model considered both the impact lag and a non-linear relationship between the exposure and response variables. The generalized linear model was selected as the basic model, and it was fitted using a quasi-Poisson regression with a log link. The independent variables were the daily mean concentrations of air pollutants and meteorological factors. The analysis also included potential confounding factors such as humidity, day of the week effects, long-term and seasonal trends, and holiday effects. The dependent variable was the number of daily hospitalizations due to respiratory system diseases. The distributed lag non-linear model is calculated by the following Eq. 2:


(2) $$\begin{array}{l} \log\left[E\left(Y_{t}\right)\right]=\alpha +\beta_{1}P_{t,l}+\beta_{2}T_{t,l}+\mathrm{NS}\left(\mathrm{time,}\;\mathrm{df}=\frac{7}{\mathrm{year}}\right)\\ +\mathrm{NS}\left(\mathrm{rh,}\;\mathrm{df}=3\right)+{\mathrm{DOW}}_{t}+{\mathrm{Holiday}}_{t}\; \end{array}$$

In this model, $Y_{t}$ represents the number of hospitalizations due to respiratory system diseases on day t, and $E\left(Y_{t}\right)$ represents the expected number of hospitalizations due to respiratory system diseases on day t. α is the intercept, $\beta_{1}$ and $\beta_{2}$ are parameter vectors, l is the lag days, $P_{t,l}$ is the cross-basis matrix for air pollutant concentrations, $T_{t,l}$ is the cross-basis matrix for temperature. The cross-basis matrix is produced by DLNM (35), the exposure-response dimension was modeled with a quadratic B-spline with 3 internal knots placed at the 10th, 75th, and 90th percentiles of air pollutants, temperature and relative humidity, and the lag-response dimension was modeled with a natural spline with an intercept and 3 internal knots equally spaced in the log scale of lag days (36–38). NS( ) is the natural cubic spline, and df is the number of cubic spline function partition that is the degree of freedom of the parameter. The variable time corresponds to the date, with a degree of freedom (df) of 7 per year for time to remove long-term trends and seasonality (39, 40). rh represents the daily relative humidity, with a degree of freedom of 3 (35, 40). ${\mathrm{DOW}}_{t}$ is dummy variable for day of the week, ${\mathrm{Holiday}}_{t}$ is holiday dummy variable.

#### 2.3.3 Analysis of multiscale geographically weighted regression model

The multiscale geographically weighted regression (MGWR) has emerged as a prominent tool for analyzing spatial heterogeneity in the relationship between variables (41–43). Compared with the traditional geographically weighted regression (GWR), the MGWR can distinguish global, regional, and local processes, permitting each variable to have a different bandwidth (44). Consequently, the MGWR model was selected in this study to explore the relationship between respiratory health and the urban built environment. It is calculated by the following Eq. 3:


(3) $$y_{i}=\overset{k}{\underset{j=1}{\sum }}\beta_{bwj}\left(u_{i},v_{i}\right)x_{ij}+\varepsilon_{i}$$

In the formula, $y_{i}$ represents the dependent variable, which is the concentration of air pollutants in a given urban block. $x_{ij}$ is the value of variable $x_{j}$ at observation point k, which includes the elevation of the urban block, the construction density, the number of facilities, or the proportion of land use. $\beta_{bwj}$ represents the local regression coefficient for the j variable with a bandwidth of bw. $\left(u_{i},v_{i}\right)$ denotes the spatial geographic location of the observation point i, and $\varepsilon_{i}$ is the error term of the model at point i.

## 3 Results

### 3.1 Descriptive statistics

The paper presents the results of a study investigating the relationship between air quality, meteorological factors, and hospitalization rates due to respiratory illnesses in the Wuhan municipal urban area. The study collected data on air pollutants, meteorological factors, and hospitalization numbers for a specific geographic location during a particular period. Table 1 provides a summary of the descriptive statistics for the collected data. The average concentration of PM_10_ (78.056 μg/m^3^) and PM_2.5_ (71.962 μg/m^3^), exceeded the first class of Chinese ambient air quality standards (2016) (Supplementary Table S1). The average concentration of PM_10_ (78.056 μg/m^3^), PM_2.5_ (71.962 μg/m^3^) and NO_2_ (54.468 μg/m^3^), exceeded the air quality guideline level of the World Health Organization (WHO) global air quality guidelines (2021) (Supplementary Table S2). SO_2_ and O_3_ concentrations were within the acceptable limits set by both organizations. The average temperature was 17.423°C, the relative humidity (RH) was 79.277%. The daily average hospitalization number was 15.97, with 9.519 for males and 6.451 for females, exhibiting a significant gender difference (Table 1). The average age of all affected persons was 54, with an average age of 57 for the male group and 48 for the female group.

**Table 1:** Descriptive statistics of air pollution, meteorological factors, and number of hospitalizations.

Variable name		Number of studied days	Maximum value	Minimum value	Average value	Standard deviation	Median value	Variance	Kurtosis	Skewness
Air Pollution	SO_2_ (μg/m^3^)	1,093	73	3	12.898	9.074	11	82.337	5.733	1.933
	NO_2_ (μg/m^3^)	1,093	125	1	54.468	22.104	52	488.566	−0.198	0.645
	PM_10_ (μg/m^3^)	1,093	474	9	78.056	40.944	70	1676.45	9.175	1.863
	O_3_ (μg/m^3^)	1,093	145	2	46.904	28.91	40	835.809	−0.274	0.692
	PM_2.5_ (μg/m^3^)	1,093	326	9	71.962	45.816	60	2099.118	2.72	1.428
Meteorol-ogical factors	Average temperature (°C)	1,093	34	−3.5	17.423	8.821	18.5	77.814	−1.169	−0.13
	RH (%)	1,093	100	41	79.277	10.776	79.75	116.132	−0.324	−0.319
Disease data	Inpatients (Pieces per day)	1,093	37	0	15.97	8.272	17	68.429	−0.935	0.003
	Male (Pieces per day)	1,093	24	0	9.519	5.144	10	26.462	−0.869	0.11
	Female (Pieces per day)	1,093	19	0	6.451	3.963	6	15.706	−0.379	0.389

SO_2_ indicates sulfur dioxide, NO_2_ indicates nitrogen dioxide, PM_10_ and PM_2.5_ indicates particulate matter, O_3_ indicates ozone, and RH indicates relative humidity.

### 3.2 Relationships between air pollutant concentrations and meteorological factors

The correlation between air pollutant concentrations and meteorological factors were examined in the following work. The analysis showed that NO_2_, PM_10_, and PM_2.5_ concentrations were positively correlated with each other (*p* < 0.01), and SO_2_ concentrations was positively correlated with NO_2_, PM_10_, and PM_2.5_ concentrations (*p* < 0.01) (Table 2). O_3_ was positively correlated with PM_10_, and negatively correlated with NO_2_ and PM_2.5_. PM_10_ and SO_2_ had the strongest correlation (*r* = 0.762, *p* < 0.01), followed by that between NO_2_ and PM_2.5_ (*r* = 0.73, *p* < 0.01) and then by that between PM_10_ and PM_2.5_ (*r* = 0.704, *p* < 0.01). Average temperature was negatively correlated with SO_2_, NO_2_, PM_10_, and PM_2.5_. Relative humidity was negatively correlated with all air pollutants. The implications of the observed correlations should be discussed further, and any limitations in the interpretation of the results should be acknowledged.

**Table 2:** Spearman correlation analysis results of air pollutant concentration with meteorological factors.

	SO_2_	NO_2_	PM_10_	O_3_	PM_2.5_	Average temperature	RH
SO_2_	1 (0.000***)					–	–
NO_2_	0.561 (0.000***)	1 (0.000***)		–		–	–
PM_10_	0.762 (0.000***)	0.656 (0.000***)	1 (0.000***)			–	–
O_3_	0.028 (0.424)	−0.143 (0.000***)	0.145 (0.000***)	1 (0.000***)	–		–
PM_2.5_	0.57 (0.000***)	0.73 (0.000***)	0.704 (0.000***)	−0.269 (0.000***)	1 (0.000***)	–	–
Average temperature	−0.393 (0.000***)	−0.354 (0.000***)	−0.256 (0.000***)	0.642 (0.000***)	−0.602 (0.000***)	1 (0.000***)	
RH	−0.368 (0.000***)	−0.221 (0.000***)	−0.411 (0.000***)	−0.404 (0.000***)	−0.178 (0.000***)	0.006 (0.84)	1 (0.000***)

SO_2_ indicates sulfur dioxide, NO_2_ indicates nitrogen dioxide, PM_10_ and PM_2.5_ indicates particulate matter, O_3_ indicates ozone, RH indicates relative humidity. ***, **, and * represent 1, 5, and 10% significance levels, respectively.

In order to test the existence of multicollinearity between the explanatory variables, SPSS26 software was used to test the covariance of each variable and obtain the Variance Inflation Factor (VIF), which showed that the VIF values of SO_2_, NO_2_, PM_10_, O_3_, PM_2.5_, average temperature and relative humidity were 1.39, 1.47, 1.26, 1.34, 1.46, 1.83, and 1.21, which are all less than 10, indicating that there is no obvious influence of multicollinearity among the explanatory variables, and the subsequent regression analysis can be carried out.

### 3.3 The relationship between air pollution, meteorological factors, and hospital admissions for respiratory diseases

We use time-series regression analysis to examine the short-term association between air pollutants (PM_10_, PM_2.5_, NO_2_, SO_2_, and O_3_), meteorological factors (average temperature and relative humidity) and respiratory diseases using distributed lag non-linear models (DLNM) with the family of Poisson distribution. Concerning previous research (45–47), a maximum lag of 7 days was assumed, and lag effects were analyzed using a triple natural cubic spline function. As temperature and humidity showed non-linear effects, a cubic spline function was utilized for their analysis. On the other hand, as air pollution exhibits linear effects, the analysis of its lag effects used a linear function. To examine the effects of temperature and humidity, cross-basis functions were used to control for these variables while also taking into account long-term and seasonal trends and day of the week effects. For air pollution, the analysis additionally controlled for temperature, humidity, long-term and seasonal trends, and day of the week effects.

**Figure 2:**
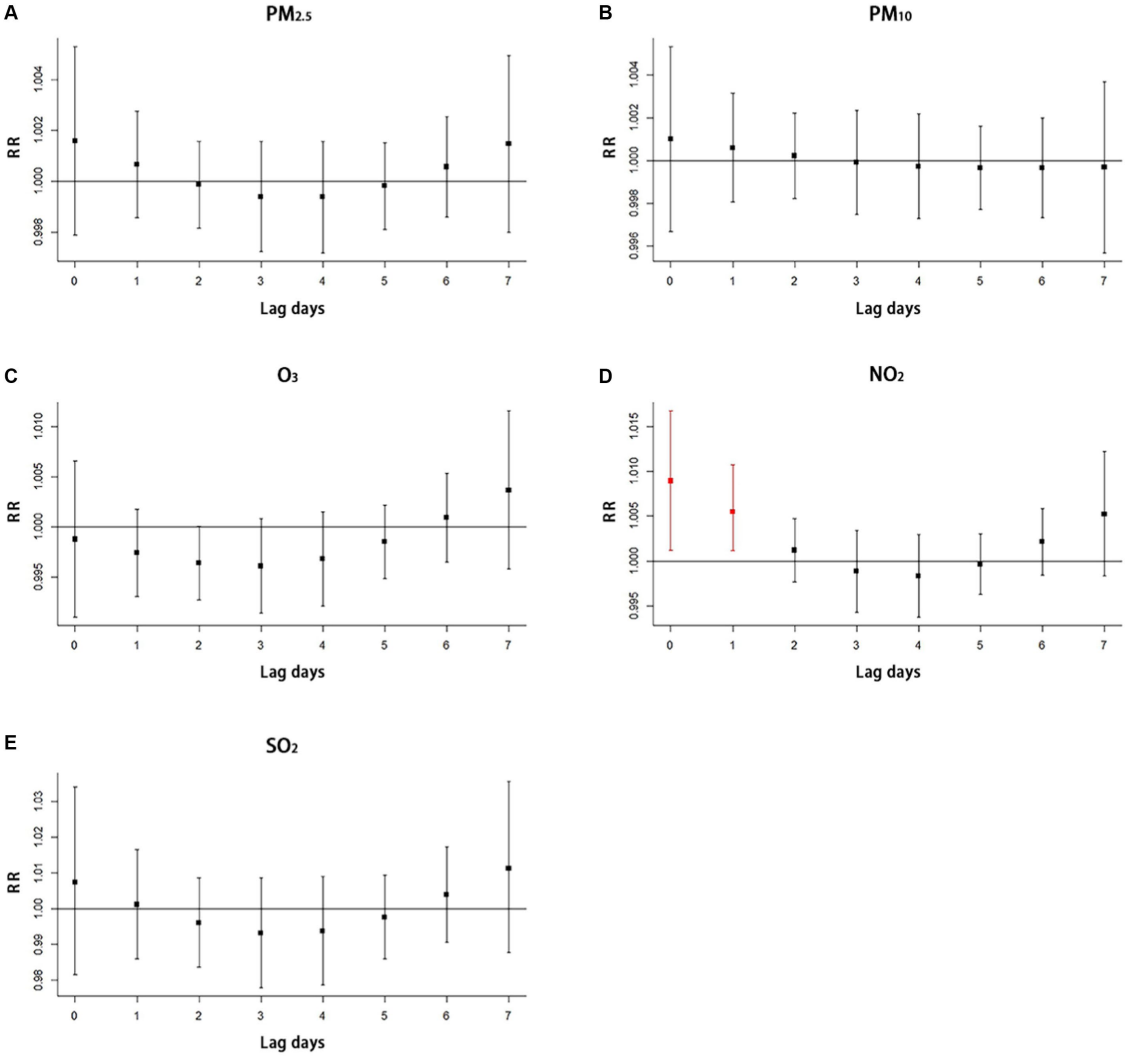
Single-lag effect of air pollutants on respiratory disease with simple exposure models **(A)** the impact of PM_2.5_ on hospitalization for respiratory diseases, **(B)** the impact of PM_10_ on hospitalization for respiratory diseases, **(C)** the impact of O_3_ on hospitalization for respiratory diseases, **(D)** the impact of NO_2_ on hospitalization for respiratory diseases, **(E)** the impact of SO_2_ on hospitalization for respiratory diseases. The red line in the figure means that statistically significant relationship was observed. SO_2_ indicates sulfur dioxide, NO_2_ indicates nitrogen dioxide, PM_10_ and PM_2.5_ indicates particulate matter, O_3_ indicates ozone, and RR indicates relative risk.

**Figure 3:**
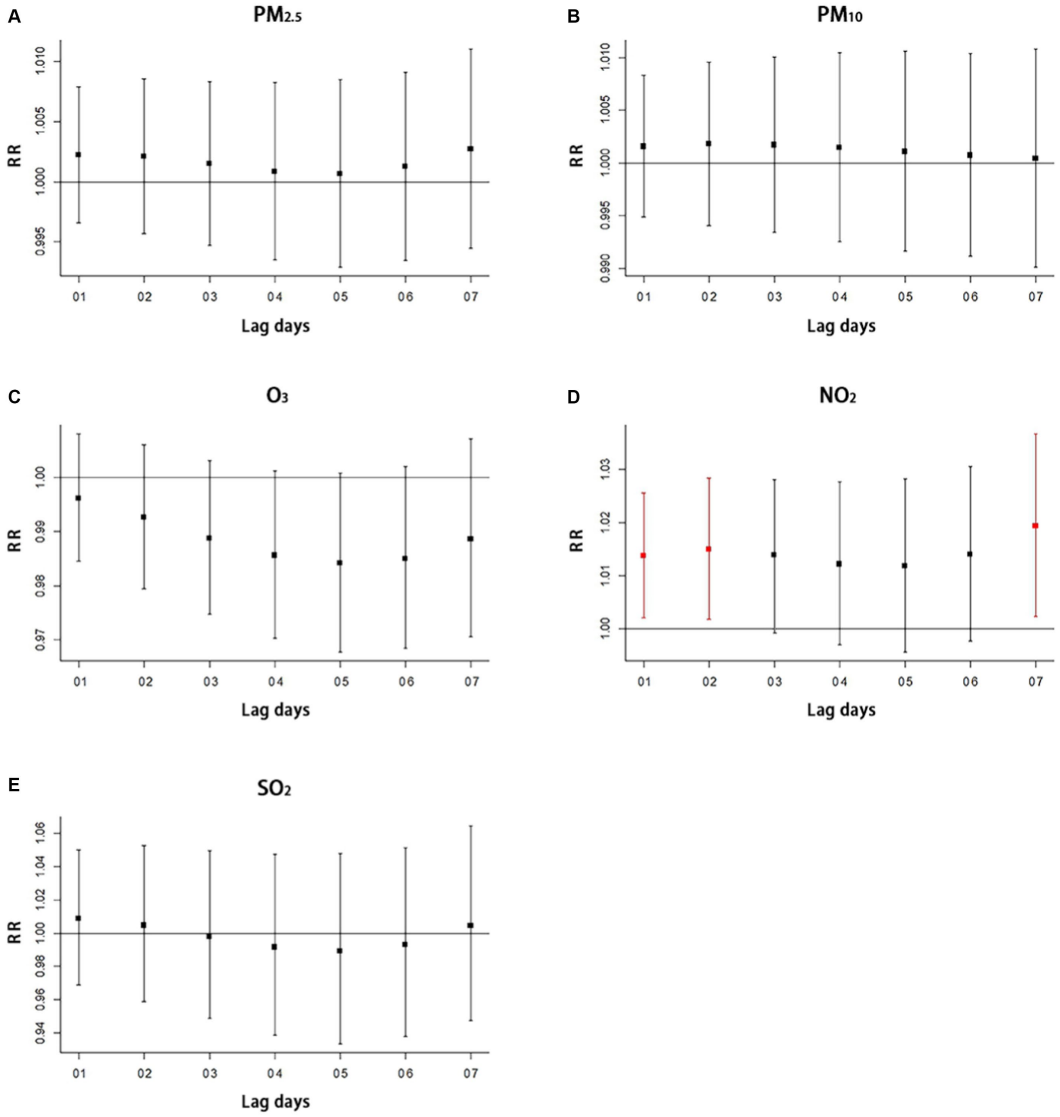
Multiple-lag effect of air pollutants on respiratory disease with cumulative exposure models **(A)** the impact of PM_2.5_ on hospitalization for respiratory diseases, **(B)** the impact of PM_10_ on hospitalization for respiratory diseases, **(C)** the impact of O_3_ on hospitalization for respiratory diseases, **(D)** the impact of NO_2_ on hospitalization for respiratory diseases, **(E)** the impact of SO_2_ on hospitalization for respiratory diseases. The 01, 02, 03, 04, 05, 06, and 07 of lag days means moving averages for the current day and the previous 1, 2, 3, 4, 5, 6, and 7 days with multiple-lag day of cumulative exposure models. The red line in the figure means that statistically significant relationship was observed. SO_2_ indicates sulfur dioxide, NO_2_ indicates nitrogen dioxide, PM_10_ and PM_2.5_ indicates particulate matter, O_3_ indicates ozone, and RR indicates relative risk.

Table 3 shows results from the single-lag day (L0–L7) of simple exposure models and multiple-lag day of cumulative exposure models (moving averages for the current day and the previous 1, 2, 3, 4, 5, 6, and 7 days: lag 01, lag 02, lag 03, lag 04, lag 05, lag 06, and lag 07) for the percent increase in respiratory diseases per 10 μg/m^3^ in air pollutants. Statistically significant relationships were observed for respiratory diseases with both 0-day lagged, and 1 day lagged (the red line in Figure 2D) of simple exposure models, 01 day lagged, 02 days lagged, and 07 days lagged (the red line in Figure 3D) of cumulative exposure models. For lag 0 and lag 1, a 10 μg/m^3^ increase in concentration of NO_2_ corresponded to 1.009% (95% CI: 1.001, 1.017%) and 1.005% (95% CI: 1.001, 1.009%) increase of respiratory diseases (Table 3). The accumulating risk of respiratory diseases increased 1.014% (95% CI: 1.002, 1.026%) at lag 01 day, 1.015% (95% CI: 1.002, 1.028%) at lag 02 days, and 1.019% (95% CI: 1.002, 1.037%) at lag 07 days with a 10 μg/m^3^ increase in concentration of NO_2_ (Table 3).

**Table 3:** Percent increase (mean and 95% CI) of daily hospital admission associated with 10 μg/m^3^ increase of pollutant concentrations in Wuhan in 2015–2017.

Lag (L)	PM_2.5_	PM_10_	SO_2_	NO_2_	O_3_
L0	1.002 (0.998, 1.005)	1.001 (0.997, 1.005)	1.007 (0.982, 1.034)	1.009 (1.001, 1.017)*	0.999 (0.991, 1.007)
L1	1.001 (0.999, 1.003)	1.001 (0.998, 1.003)	1.001 (0.986, 1.017)	1.005 (1.001, 1.011)*	0.997 (0.993, 1.002)
L2	1 (0.998, 1.002)	1 (0.998, 1.002)	0.996 (0.984, 1.009)	1.001 (0.998, 1.005)	0.996 (0.993, 1)
L3	0.999 (0.997, 1.002)	1 (0.997, 1.002)	0.993 (0.978, 1.009)	0.999 (0.994, 1.003)	0.996 (0.991, 1.001)
L4	0.999 (0.997, 1.002)	1 (0.997, 1.002)	0.994 (0.979, 1.009)	0.998 (0.994, 1.003)	0.997 (0.992, 1.002)
L5	1 (0.998, 1.002)	1 (0.998, 1.002)	0.998 (0.986, 1.009)	1.01 (0.996, 1.003)	0.999 (0.995, 1.002)
L6	21.001 (0.999, 1.003)	1 (0.997, 1.002)	1.004 (0.991, 1.017)	1.002 (0.998, 1.006)	1.001 (0.996, 1.005)
L7	1.001 (0.998, 1.005)	1 (0.996, 1.004)	1.011 (0.988, 1.036)	1.005 (0.998, 1.012)	1.004 (0.996, 1.012)
L01	1.002 (0.997, 1.008)	1.002 (0.995, 1.008)	1.009 (0.969, 1.05)	1.014 (1.002, 1.026)*	0.996 (0.985, 1.008)
L02	1.002 (0.996, 1.009)	1.002 (0.994, 1.01)	1.005 (0.959, 1.053)	1.015 (1.002, 1.028)*	0.993 (0.979, 1.006)
L03	1.001 (0.995, 1.008)	1.002 (0.993, 1.01)	0.998 (0.949, 1.049)	1.014 (0.999, 1.028)	0.989 (0.975, 1.003)
L04	1.001 (0.994, 1.008)	1.001 (0.993, 1.01)	0.991 (0.938, 1.047)	1.012 (0.997, 1.028)	0.986 (0.97, 1.001)
L05	1.001 (0.993, 1.009)	1.001 (0.992, 1.011)	0.989 (0.934, 1.048)	1.012 (0.996, 1.028)	0.984 (0.968, 1.001)
L06	1.001 (0.993, 1.009)	1.001 (0.991, 1.01)	0.993 (0.938, 1.052)	1.014 (0.998, 1.031)	0.985 (0.968, 1.002)
L07	1.003 (0.994, 1.011)	1 (0.99, 1.011)	1.004 (0.947, 1.064)	1.019 (1.002, 1.037)*	0.989 (0.971, 1.007)

Lag indicates the lag between air pollutant and occurrence of respiratory diseases. SO_2_ indicates sulfur dioxide, NO_2_ indicates nitrogen dioxide, PM_10_ and PM_2.5_ indicates particulate matter, O_3_ indicates ozone. *Statistically significant (*p* < 0.05).

The study did not find significant lag effects for O_3_ (Figures 2C, 3C), PM_2.5_ (Figures 2A, 3A), PM_10_ (Figures 2B, 3B), and SO_2_ (Figures 2E, 3E) with both simple exposure models and cumulative exposure models. No correlations were observed for average temperature and relative humidity (Supplementary Figures S1, S2).

### 3.4 Impact of built environment on air pollution

#### 3.4.1 Spatial distribution characteristics of built environment elements

**Figure 4:**
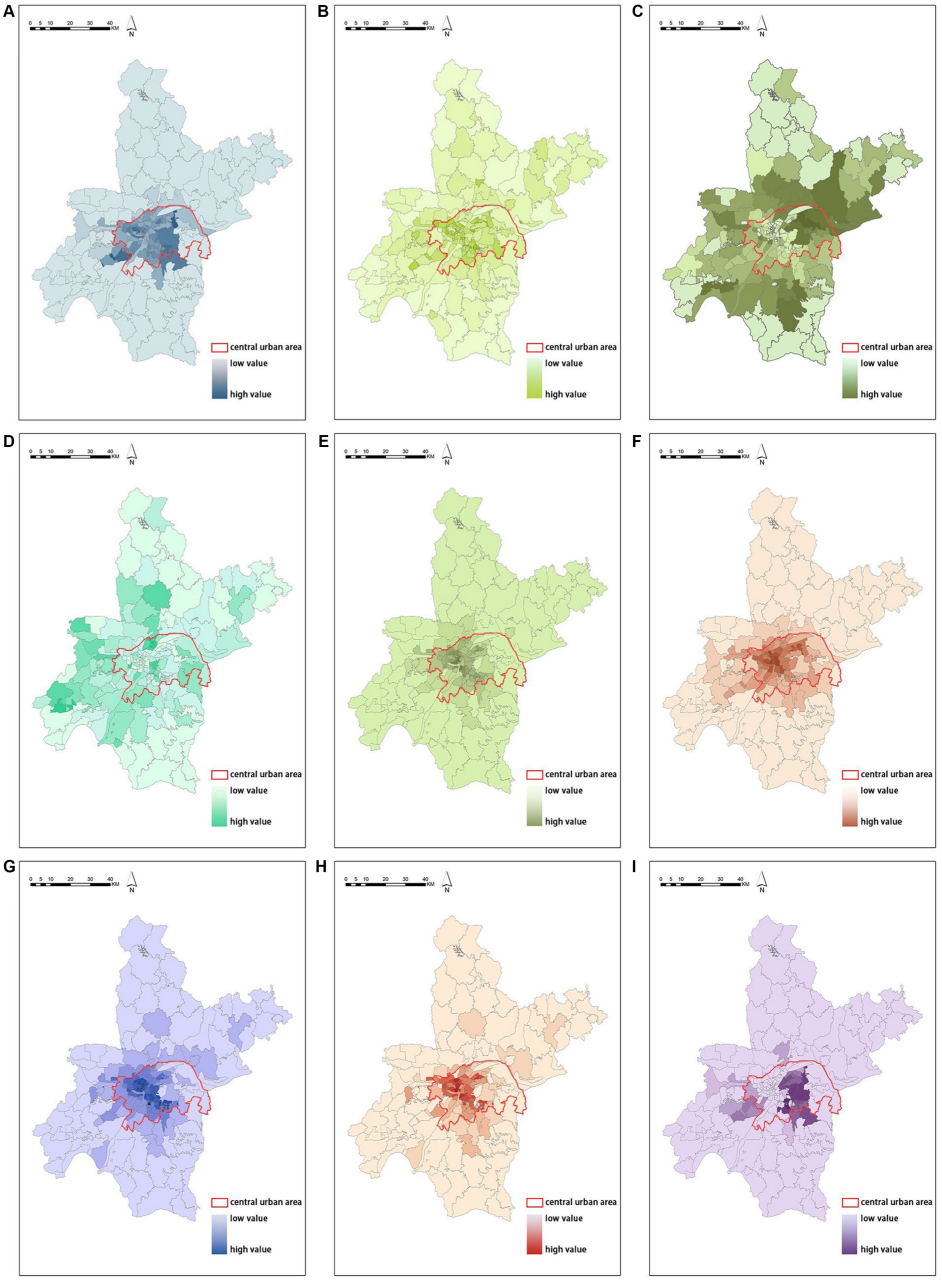
Spatial distribution of built environment **(A)** spatial distribution of building density, **(B)** spatial distribution of road density, **(C)** spatial distribution of the percentage of industrial land, **(D)** spatial distribution of the percentage of park and green space, **(E)** spatial distribution of the number of shopping service facilities, **(F)** spatial distribution of the percentage of construction land, **(G)** spatial distribution of quantity of the number of business residences, **(H)** spatial distribution of population density, **(I)** spatial distribution of plot ratio.

The following figures show the spatial distribution characteristics of the built environment elements in Wuhan city (Figures 4A–I). It is apparent that the building density in the central urban area is generally much higher than that in the peripheral suburbs. The road density, percentage of construction land, plot ratio, number of business residences and shopping service facilities show apparent spatial polarization, portraying a core-edge distribution pattern centered on the central city. Higher percentage of industrial land in the eastern, western and southern regions compared to the northern and central regions. Compared to other parameters, the spatial distribution of park and green land ratio appears moderately balanced, with lower percentages allocated towards edge areas.

#### 3.4.2 Spatial distribution characteristics of air pollution

**Figure 5:**
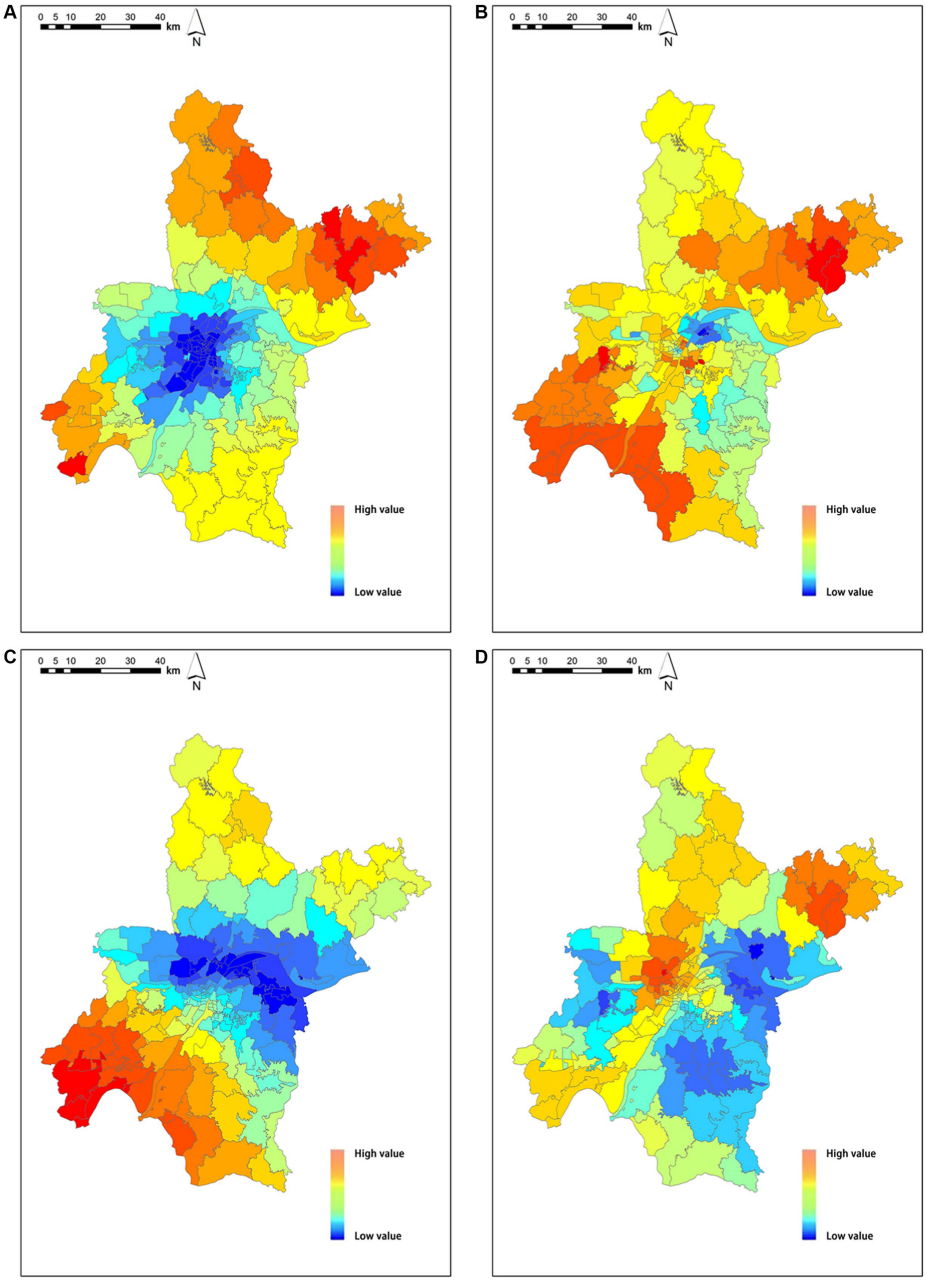
Air pollution concentration spatial distribution **(A)** spatial distribution of NO_2_, **(B)** spatial distribution of PM_2.5_, **(C)** spatial distribution of PM_10_, **(D)** spatial distribution of SO_2_. SO_2_ indicates sulfur dioxide, NO_2_ indicates nitrogen dioxide, PM_10_ and PM_2.5_ indicates particulate matter.

The following figures show the spatial distribution of NO_2_ (Figure 5A), PM_2.5_ (Figure 5B), PM_10_ (Figure 5C), and SO_2_ (Figure 5D). NO_2_ concentrations are clustered with low values in the central region, high values in the northern region, and dominated by medium values in the southern region; the medium and high values of PM_2.5_ concentrations are mainly distributed in the south and north, with more low and medium values in the central region; PM_10_ concentrations are clustered with low values in the central region, high values in the southern region, and dominated by medium values in the northern region; and SO_2_ concentrations are clustered with low values in the eastern and western regions, dominated by medium values in the central region and high values predominate in the central region.

#### 3.4.3 Built environment’s spatial heterogeneity impact on the distribution of air pollutants

Based on the results of the distribution lag non-linear model, our study only observed a significant lag effect of NO_2_ on respiratory diseases. Therefore, in the MGWR analysis, only NO_2_ was analyzed to investigate the spatial heterogeneity effects of the built environment on NO_2_ distribution.

As shown in Table 4, the goodness of adjust *R*^2^ of multiscale geographically weighted regression (MGWR) is greater than the geographically weighted regression (GWR), and the value of AICc is lower than the GWR model, indicating that the MGWR model has better regression results. MGWR has a smaller number of effective parameters and residual sum of squares (RSS) than the GWR model, indicating that the MGWR model obtains regression results closer to the true values using fewer parameters (Table 4).

**Table 4:** Geographically weighted regression (GWR) versus multiscale geographically weighted regression (MGWR) model metrics.

Model indicators	GWR	MGWR
Residual sum of squares (RSS)	64.891	3.550
Effective number of parameters	69.3	55.272
Log-likelihood	−165.6	103.189
Akaike information criterion (AICc)	357.007	−43.372
*R* ^2^	0.649	0.981
Adjust *R*^2^	0.629	0.973

GWR indicates geographically weighted regression model, MGWR indicates multiscale geographically weighted regression model.

Table 5 displays the bandwidths of the MGWR model, which utilizes adaptive bandwidths to show differences in the spatial operating scales of each independent variable (48) (Table 5). In this study, there are 185 block units, and the explanatory variables employed are not at a general scale, indicating the variance in spatial heterogeneity operating scales. The MGWR model has the optimum bandwidth at 43, with smaller bandwidths for population density, building density, number of shopping service facilities, and construction land. These smaller bandwidths indicate that these four variables have a greater impact on spatial heterogeneity in NO_2_.

**Table 5:** Multiscale geographically weighted regression (MGWR) model bandwidths.

Variable	Bandwidth
Intercept	43
Population density	43
Building density	43
Plot ratio	75
Road density	58
Number of business residences	44
Number of shopping service facilities	43
Construction land	43
Percentage of industrial land	59
Percentage of park and green space	66

As shown in Table 6, the percentage of park and green space showed a significant negative correlation with NO_2_ concentration with regression coefficients of −0.064. Intercept, population density, building density, plot ratio, road density, number of business residences, number of shopping service facilities, construction land, and percentage of industrial land were significantly positively correlated with NO_2_ concentration, and their regression coefficients were 0.691, 0.199, 0.202, 0.016, 0.013, 0.187, 0.273, 0.249, and 0.102. In terms of the magnitude of the absolute value of the regression coefficients, number of shopping service facilities (exclude intercept) was the main driver of the change in NO_2_ concentration, followed by construction land, building density and other variables.

**Table 6:** Summary statistics of fit coefficients for multiscale geographically weighted regression (MGWR) parameters.

Variable	Mean	Standard deviation	Minimal	Median	Maximum
Intercept	0.691	0.226	0.238	0.747	0.96
Population density	0.199	0.208	−0.001	0.118	0.665
Building density	0.202	0.211	−0.021	0.118	0.626
Plot ratio	0.016	0.07	−0.067	−0.016	0.311
Road density	0.013	0.074	−0.063	−0.004	0.311
Number of business residences	0.187	0.24	−0.049	0.063	0.75
Number of shopping service facilities	0.273	0.3	−0.026	0.158	0.916
Construction land	0.249	0.195	0.048	0.168	0.645
Percentage of industrial land	0.102	0.073	−0.064	−0.035	0.228
Percentage of park and green space	−0.064	0.077	−0.22	−0.036	0.151

**Figure 6:**
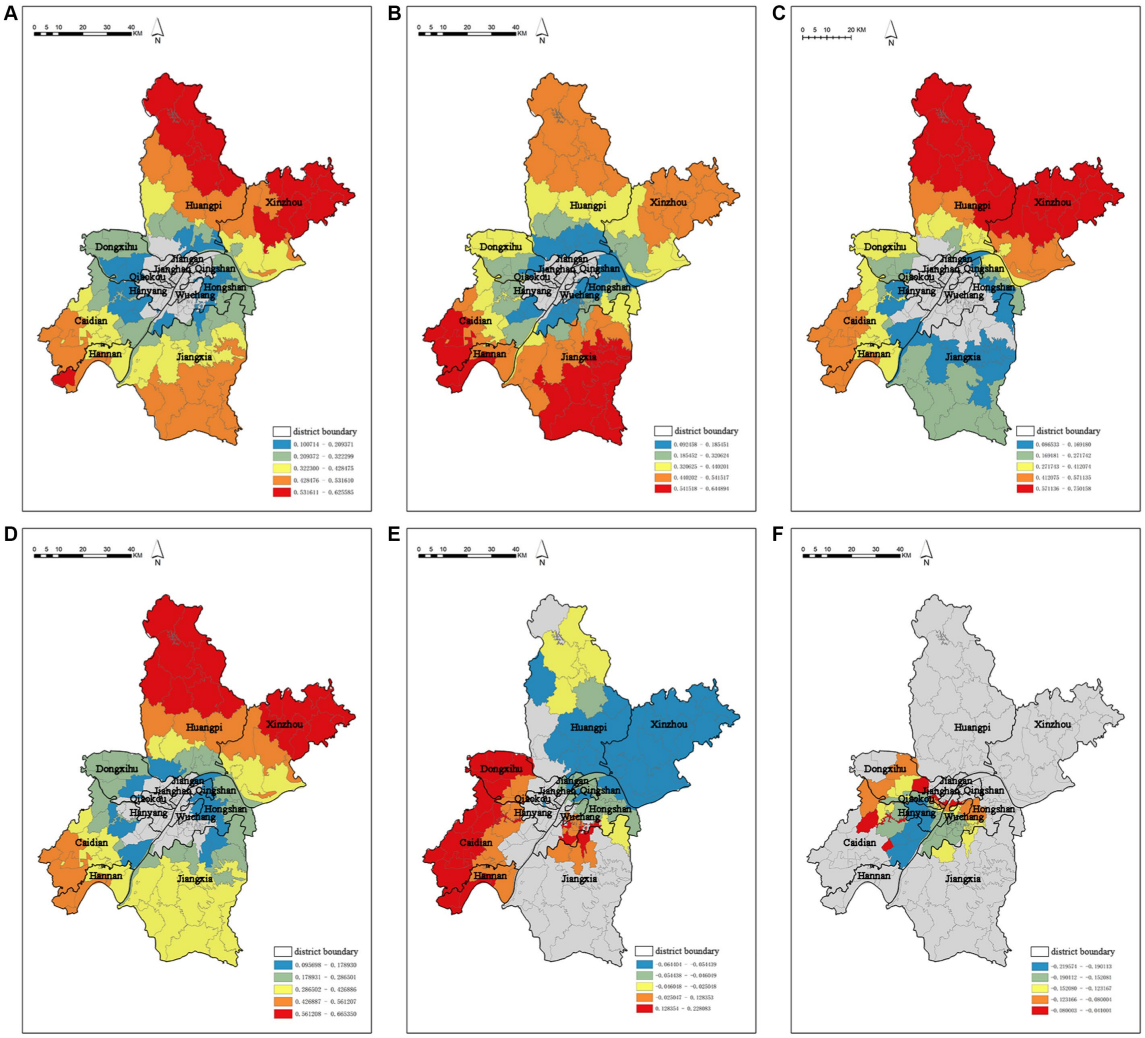
Spatial coefficients distribution of driving factors based on the MGWR model **(A)** spatial coefficient distribution of building density, **(B)** spatial coefficient distribution of construction land, **(C)** spatial coefficient distribution of the number of business residences, **(D)** spatial coefficient distribution of population density, **(E)** spatial coefficient distribution of the percentage of industrial land, **(F)** spatial coefficient distribution of the percentage of park and green space.

The following figures display the spatial heterogeneity effects of NO_2_ concentration and air pollution (Figures 6A–F). Based on the analysis above, further analysis was conducted on the typical and significant findings. Capacity coefficients with *p*-values greater than 0.05 were eliminated, and visual analysis was carried out using the ArcGIS platform. Subsequently, the spatial coefficient distribution results of the significant driving factors were further screened.

It is worth mentioning that MGWR not only reflects the multi-scale spatial effects of air pollution concentration factors, but also identifies statistically insignificant spatial units. Building density (Figure 6A), construction land (Figure 6B), number of business residences (Figure 6C), and population density (Figure 6D) do not show significant characteristics in the central urban area. The remaining driving factors show strong spatial stratification heterogeneity from the central to the peripheral areas. This suggests that the impact of the urban built environment on air pollution concentration is not significant within the central urban area, but it has a good correlation in a broader range. Percentage of industrial land and percentage of park and green space have a significant impact on the central urban area.

The capacity coefficient of building density ranges from 0.100714 to 0.625585, and it has a significant positive effect on the spatial distribution of NO_2_ concentration (Figure 6A). The high-value areas for building density are mainly concentrated in the Huangpi District and Xinzhou District in the north, while the low-value areas are mainly distributed in the northern Caidian District, southern Dongxihu District, and Hongshan District on the edge of the central urban area. Similarly, the capacity coefficient of construction land ranges from 0.092458 to 0.644894 and also has a significant positive effect on the spatial distribution of NO_2_ concentration (Figure 6B). The high-value areas are mainly distributed in Jiangxia District, Caidian District, and Hannan District, while the low-value areas are distributed in the peripheral areas of the central urban area. The capacity coefficients of number of business residences (Figure 6C) and population density (Figure 6D) range from 0.086533 to 0.750158 and 0.095698 to 0.665350, respectively, and they both have a significant positive effect on the spatial distribution of NO_2_ concentration. The high-value areas are mainly located in the Huangpi District and Xinzhou District in the north, while the low-value areas exhibit a scattered distribution around the edge of the central urban area.

The percentage of industrial land (Figure 6E) have spatial heterogeneity impacts on the distribution of NO_2_ concentration. The capacity coefficient of percentage of industrial land ranges from −0.064404 to 0.228083, demonstrating a strong spatial unevenness. It shows a significant promotion effect on NO_2_ concentration in Qing Shan District, Hannan District, and Caidian District, while exhibiting a strong negative correlation in Xinzhou District. The capacity coefficient of percentage of park and green space (Figure 6F) ranges from −0.219574 to −0.041001, and its influence mainly radiates within the central urban area, including Wuchang District, Jiangan District, and Jianghan District.

## 4 Discussion

This study analyzed the cascading mechanism of “urban built environment-air pollution-respiratory diseases,” then the study was divided into several specific experimental steps. Firstly, the correlation analysis of the study found that meteorological factors (average temperature, relative humidity) had a significant effect on the concentration of air pollutants (PM_2.5_, PM_10_, SO_2_, NO_2_, O_3_). Average temperature and relative humidity showed a significant negative correlation with air pollutants (PM_2.5_, PM_10_, SO_2_, NO_2_). Average temperature showed a significant positive correlation with O_3_, relative humidity showed a significant negative correlation with O_3_. Average temperature and relative humidity are important meteorological factors that affect the generation, transport and dispersion of air pollutants (49), they can influence the dispersion and concentration of air pollutants by affecting the vertical mixing of air (50). Wuhan is surrounded by mountains on three sides, with poor air circulation, and has a large number of lakes and rivers. All these factors contribute to the humid and windy climate characteristics of Wuhan, which are not conducive to the dispersion of air pollutants (51).

We observed only the lagged effect of NO_2_ on respiratory diseases through the analysis of distributed lag non-linear model. When lagged 0 day and 1 day, a 10 μg/m^3^ increase in NO_2_ concentration corresponded to 1.009% (95% CI: 1.001, 1.017%) and 1.005% (95% CI: 1.001, 1.011%) increase of hospital admission for respiratory diseases. The lagged effect of NO_2_ on respiratory diseases has already been demonstrated in previous studies. A study in Shanghai, China, showed that when lagged for 5 days, a 10 μg/m^3^ increase in NO_2_ concentration corresponded to 0.65% (95% CI: −0.37, 1.68%) increase in hospital admissions for respiratory diseases in the cold season and 0.05% (95% CI: −0.91, 1.02%) increase in the warm season (52). As for PM_2.5_, PM_10_, and SO_2_, no lagged effect was observed, despite showing a correlation with respiratory diseases. This is not the same as previous studies. A study from Dongguan, China, showed that at a lag of 3 days, an IQR (interquartile range) increase in PM_2.5_ was associated with a 15.41% (95% CI: 10.99, 20.01%) increase in respiratory morbidity (8). A study from Shanghai, China showed that when lagged by 5 days, a 10 μg/m^3^ increase in PM_10_ and SO_2_ concentrations corresponded to 0.09% (95% CI: −0.25, 0.43%), 0.65% (95% CI: 0.04, 1.25%) increase in hospital admissions for respiratory diseases in the cold season and 0.13% (95% CI: −0.30, 0.57%), 0.24% (95% CI: −0.56, 1.03%) in the warm season (52). For O_3_, the study did not find a correlation with respiratory disease.

The study analyzed the spatial heterogeneity effects of built environment elements on NO_2_ distribution using the MGWR model. We observed that building density, construction land, number of business residences and population density do not have a significant effect on NO_2_ in the central urban area, but show a stratified spatial heterogeneity effect from the periphery of the central urban area to the suburbs. The impact of building density, construction land, and number of business residences on air pollutants comes from many sources, including building energy consumption (53, 54), air pollutants emitted by restaurants and recreational facilities (55, 56), and vehicle emission (54, 57). Spatial changes in population density can also directly affect transportation travel, food consumption, and manufacturing in different areas, which in turn affects the concentration of air pollutants (58, 59). Consequently, rather than concentrating just on the downtown region, Wuhan’s future public health strategies should take the optimization of environmental health throughout the entire city into account. And percentage of industrial land has a significant positive effect on NO_2_ in the central urban area, which is consistent with the actual situation in Wuhan. Qingshan District in the center of Wuhan is an important old industrial base in Wuhan with a large number of industrial plants; Caidian District has many manufacturing factories led by Dongfeng Automobile Company; similarly, Hannan District is an important automobile manufacturing and industrial production base in Wuhan. These factors largely strengthen the contribution of percentage of industrial land to NO_2_ in these areas. Percentage of park and green space has a significant negative effect on NO_2_ in the central urban area, which is closely related to the large number of parks and green spaces in the central urban areas, such as Wuchang, Jiangan, and Jianghan Districts. This is closely related to the large number of parks and green spaces in these districts. By analyzing the results of the MGWR model, the spatial heterogeneity of NO_2_ distribution by built environment elements are, in descending order, as follows: population density, building density, construction land, number of shopping service facilities, number of business residences, road density, percentage of industrial land, percentage of park and green space, plot ratio.

Through a methodical examination of every aspect of the built environment in our investigation, we discovered that the effects of built environment elements on NO_2_ showed strong heterogeneity in the central and marginal areas. Accordingly, there is no doubt that urban development polarizes the city’s center and periphery’s air quality by contributing to the spread of NO_2_. Parks and green spaces certainly are one of the best means to enhance air quality and alleviate air pollution in high-density urban environments, this has been confirmed many times in previous studies (60–62).

It is also important to note that this study practically investigated the cascading mechanism of “urban built environment-air pollution- respiratory diseases,” and broken it down into specific experimental steps. The built environment is a significant influencing element that has been largely ignored in previous public health research, which has mostly examined the effect of air pollution on respiratory disorders alone (3, 4, 6). Of course, there are scholars from the field of urban studies who have analyzed the impact of built environment elements on air pollutants (22), but there is a lack of correlation analysis with respiratory diseases. Furthermore, some researchers have examined the direct relationship between respiratory illnesses and the built environment (18), but these studies have overlooked the significant impact that air pollution plays as an intermediate component. This study successfully combines the above three factors and analyzes the influential relationship between them. Even though this approach is still in its infancy, it can yet yield fresh concepts for investigations down the road.

Of course, this study has some limitations. First, the health data used in this study came from one hospital, which is hardly representative of respiratory diseases in the whole city. Second, this study did not group the population according to age, gender, type of disease, education level, etc., and could not give effective suggestions for different populations. In addition, due to the difficulty of obtaining data, this study divides the units into streets and counts the spatial distribution of built environment elements and air pollutants. This way of dividing the units inevitably has some deviation from the actual situation, and a more detailed and scientific way needs to be researched after collecting more detailed data. This study only analyzed typical built environment elements, and it is difficult to include all built environment elements. There are other types of built environment factors that also affect the distribution of air pollutants, such as building ventilation rate (63). All of these elements are expected to be analyzed and explored in detail in our follow-up studies. Finally, although studies have confirmed that built environment elements have an impact on respiratory diseases (18), this study did not analyze whether built environment elements have a direct impact on respiratory diseases due to the limitation of various factors such as the length of the article, because it has been observed in our previous study (30). Accordingly, we will improve the cascading mechanism by utilizing more adequate data and more scientific statistical methods to realize this experimental step in our subsequent studies.

## 5 Conclusion

In this study, we analyzed the cascading mechanism of “urban built environment-air pollution-respiratory diseases” through four specific experimental steps. Distinguishing from previous studies that focused only on the relationship between two factors, we analyzed the cascading influence relationship between three factors: built environment, air pollution, and respiratory diseases. We found a lagged effect of NO_2_ on respiratory diseases, when lagged 0 day and 1 day, a 10 μg/m^3^ increase in NO_2_ concentration corresponded to 1.009% (95% CI: 1.001, 1.017%) and 1.005% (95% CI: 1.001, 1.011%) increase of hospital admission for respiratory diseases. Also, we observed that building density, construction land, number of business residences and population density exhibited spatial heterogeneity on NO_2_ distribution in the central urban and suburban areas. In the central urban area, percentage of industrial land had a significant contribution to NO_2_, while the percentage of park and green space had a significant inhibitory effect on NO_2_.

Based on the effects of this spatial heterogeneity found in our study, we propose that future healthy city planning and public health policy development needs to expand the scope of consideration to include urban and rural spaces as a whole, rather than building healthy urban centers. When public health policies consider mitigating respiratory diseases by curbing air pollution, they also need to take into account the impact of built environment elements on air pollution; focusing on air pollution alone cannot mitigate respiratory diseases at the source. Therefore, in the future, the public health sector needs to work together with the urban construction sector, natural resources sector and ecological environment sector when formulating health policies. Through multi-sectoral cooperation to optimize the urban built environment, alleviate the impact of air pollution, so as to create a healthier urban respiratory environment.

## Data availability statement

The original contributions presented in the study are included in the article/Supplementary material, further inquiries can be directed to the corresponding authors.

## Author contributions

ZZ: Conceptualization, Data curation, Formal analysis, Methodology, Project administration, Visualization, Software, Writing – original draft, Writing – review & editing. YD: Conceptualization, Methodology, Visualization, Software, Writing – original draft. RG: Data curation, Investigation, Visualization, Software, Writing – review & editing. QW: Conceptualization, Methodology, Project administration, Resources, Writing – original draft. YJ: Methodology, Supervision, Project administration, Funding acquisition, Writing – review & editing.

## Footnotes

^1^ https://hbj.wuhan.gov.cn/

## References

[1] Brunekreef B Holgate ST. Air pollution and health. Lancet. (2002) 360:1233–42. doi: 10.1016/S0140-6736(02)11274-8 12401268

[2] Samet J Krewski D. Health effects associated with exposure to ambient air pollution. J Toxic Environ Health A. (2007) 70:227–42. doi: 10.1080/15287390600884644 17365585

[3] Tsai SS Chiu HF Liou SH Yang CY. Short-term effects of fine particulate air pollution on hospital admissions for respiratory diseases: a case-crossover study in a Tropical city. J Toxic Environ Health A. (2014) 77:1091–101. doi: 10.1080/15287394.2014.922388 25072896

[4] Tsangari H Paschalidou AK Kassomenos AP Vardoulakis S Heaviside C Georgiou KE. Extreme weather and air pollution effects on cardiovascular and respiratory hospital admissions in Cyprus. Sci Total Environ. (2016) 542:247–53. doi: 10.1016/j.scitotenv.2015.10.106 26519584

[5] Najm AM Carpenter DO. Patterns of emergency room visits for respiratory diseases in New York state in relation to air pollution, poverty and smoking. Int J Environ Res Public Health. (2023) 20:3267. doi: 10.3390/ijerph20043267 36833962 PMC9966596

[6] Stieb DM Szyszkowicz M Rowe BH Leech JA. Air pollution and emergency department visits for cardiac and respiratory conditions: a multi-city time-series analysis. Environ Health. (2009) 8:25. doi: 10.1186/1476-069X-8-25 19515235 PMC2703622

[7] Kousha T Rowe BH. Ambient ozone and emergency department visits due to lower respiratory condition. Int J Occup Med Environ Health. (2014) 27:50–9. doi: 10.2478/s13382-014-0229-0 24464442

[8] Zhao YJ Wang SY Lang LL Huang CY Ma WJ Lin HL. Ambient fine and coarse particulate matter pollution and respiratory morbidity in Dongguan, China. Environ Pollut. (2017) 222:126–31. doi: 10.1016/j.envpol.2016.12.070 28041838

[9] Liu WL Xu ZP Yang TN. Health effects of air pollution in China. Int J Environ Res Public Health. (2018) 15:1471. doi: 10.3390/ijerph15071471 30002305 PMC6068713

[10] Guo YM Jia YP Pan XC Liu LQ Wichmann HE. The association between fine particulate air pollution and hospital emergency room visits for cardiovascular diseases in Beijing, China. Sci Total Environ. (2009) 407:4826–30. doi: 10.1016/j.scitotenv.2009.05.022 19501385

[11] Liang LR Cai YT Barratt B Lyu BL Chan Q Hansell AL. Associations between daily air quality and hospitalisations for acute exacerbation of chronic obstructive pulmonary disease in Beijing, 2013–17: an ecological analysis. Lancet Planet Health. (2019) 3:e270–9. doi: 10.1016/S2542-5196(19)30085-3 31229002 PMC6610933

[12] Liu JY Li YF Li J Liu Y Tao NN Song WM. Association between ambient PM_2.5_ and children’s hospital admissions for respiratory diseases in Jinan, China. Environ Sci Pollut Res. (2019) 26:24112–20. doi: 10.1007/s11356-019-05644-7 31228058

[13] Dong JY Wang YR Wang JC Bao HR. Association between atmospheric PM_2.5_ and daily outpatient visits for children’s respiratory diseases in Lanzhou. Int J Biometeorol. (2021) 65:989–99. doi: 10.1007/s00484-021-02080-6 33587184

[14] Dong JY Yang RQ Zhai GY Wang JC Bao HR. Risks of hospital outpatient visits for overall and cause-specific respiratory disease associated with particulate matter pollution in Lanzhou, China. Air Qual Atmos Health. (2021) 14:1405–15. doi: 10.1007/s11869-021-01030-w

[15] Venners SA Wang BY Peng ZG Xu Y Wang LH Xu XP. Particulate matter, sulfur dioxide, and daily mortality in Chongqing, China. Environ Health Perspect. (2003) 111:562–7. doi: 10.1289/ehp.5664 12676616 PMC1241445

[16] Xia CX Ma JD Wang J Huang J Shen Q Chen YL. Quantification of the exposure–lag–response association between air pollution and respiratory disease morbidity in Chongqing city, China. Environ Model Assess. (2019) 24:331–9. doi: 10.1007/s10666-018-9625-3

[17] Wang L Chen R Sun WY Yang XM Li XH. Impact of high-density urban built environment on chronic obstructive pulmonary disease: a case study of Jing’an district, Shanghai. Int J Environ Res Public Health. (2020) 17:252. doi: 10.3390/ijerph17010252 PMC6982330 31905874

[18] Witri I Muhammad HY Prani S Djoni H. The relationship between the built environment and respiratory health: evidence from a longitudinal study in Indonesia. SSM – Popul Health. (2022) 19:101193. doi: 10.1016/j.ssmph.2022.101193 36105559 PMC9464964

[19] Li S Zhu L Shi TM Wang W. Planning strategies for PM pollution prevention and control in urban neighborhoods. Urban Dev Res. (2014) 21:42–5. doi: 10.3969/j.issn.1006-3862.2014.01.007

[20] Weng QH Yang SH. Urban air pollution patterns, land use, and thermal landscape: an examination of the linkage using GIS. Environ Monit Assess. (2006) 117:463–89. doi: 10.1007/s10661-006-0888-9 16917724

[21] Schweitzer L Zhou JP. Neighborhood air quality, respiratory health, and vulnerable populations in compact and sprawled regions. J Am Plan Assoc. (2010) 76:363–71. doi: 10.1080/01944363.2010.486623

[22] Sun SP Gu RJ Zhang J. Different greenery coverage and green space types and airborne respirable particulate matter (PM_10_) in urban areas of Beijing. China Garden. (2004) 3:80–2. doi: 10.3969/j.issn.1000-6664.2004.03.022

[23] Li F. Study on the influence of built environment on urban PM_2.5_ concentration and its management at multiple scales Chongqing University (2022).

[24] Wang L Zhao XJ Jiang XJ Tang J. Research on healthy city planning from the perspective of particulate matter distribution--theoretical framework and empirical method. Urban Plan. (2016) 40:39–48. doi: 10.11819/cpr20160906a

[25] Sun YL Zhuang GS Ying W Han LH Guo JH Mo D. The air-borne particulate pollution in Beijing—concentration, composition, distribution and sources. Atmos Environ. (2004) 38:5991–6004. doi: 10.1016/j.atmosenv.2004.07.009

[26] Ho CC Chan CC Cho CW Lin HI Lee JH Wu CF. Land use regression modeling with vertical distribution measurements for fine particulate matter and elements in an urban area. Atmos Environ. (2015) 104:256–63. doi: 10.1016/j.atmosenv.2015.01.024

[27] Xu H Bi XH Zheng WW Wu JH Feng YC. Particulate matter mass and chemical component concentrations over four Chinese cities along the western Pacific coast. Environ Sci Pollut Res. (2015) 22:1940–53. doi: 10.1007/s11356-014-3630-0 25292296

[28] Zhan DS Zhang QY Xu XR Zeng CS. Spatiotemporal distribution of continuous air pollution and its relationship with socioeconomic and natural factors in China. Int J Environ Res Public Health. (2022) 19:6635. doi: 10.3390/ijerph19116635 35682220 PMC9180089

[29] Liang S Sun C Liu CF Jiang LL Xie YJ Yan SH. The influence of air pollutants and meteorological conditions on the hospitalization for respiratory diseases in Shenzhen city, China. Int J Environ Res Public Health. (2021) 18:5120. doi: 10.3390/ijerph18105120 34065982 PMC8151817

[30] Jia YF Zhang ZQ Ding Y. Research on the influence of neighborhood environment on respiratory health and its optimization. Landscape Archit. (2023) 30:40–8. doi: 10.12409/j.fjyl.202304100176

[31] Gong P Liu H Zhang MN Li CC Wang J Huang HB. Stable classification with limited sample: transferring a 30-m resolution sample set collected in 2015 to mapping 10-m resolution global land cover in 2017. Sci Bull. (2019) 64:370–3. doi: 10.1016/j.scib.2019.03.002 36659725

[32] Stockfelt L Andersson EM Molnár P Gidhagen L Segersson D Rosengren A. Long-term effects of total and source-specific particulate air pollution on incident cardiovascular disease in Gothenburg, Sweden. Environ Res. (2017) 158:61–71. doi: 10.1016/j.envres.2017.05.036 28600978

[33] Kim J Kim H. Influence of ambient temperature and diurnal temperature range on incidence of cardiac arrhythmias. Int J Biometeorol. (2017) 61:407–16. doi: 10.1007/s00484-016-1221-0 27568189

[34] Cui LJ Geng XY Ding T Tang J Xu JX Zhai JX. Impact of ambient temperature on hospital admissions for cardiovascular disease in Hefei city, China. Int J Biometeorol. (2019) 63:723–34. doi: 10.1007/s00484-019-01687-0 30852664

[35] Gasparrini A Armstrong B Kenward MG. Distributed lag non-linear models. Stat Med. (2010) 29:2224–34. doi: 10.1002/sim.3940 20812303 PMC2998707

[36] Alahmad B Khraishah H Roye D Vicedo-Cabrera AM Guo YM Papatheodorou SI. Associations between extreme temperatures and cardiovascular cause-specific mortality: results from 27 countries. Circulation. (2023) 147:35–46. doi: 10.1161/CIRCULATIONAHA.122.061832 36503273 PMC9794133

[37] Zhao Q Guo YM Ye TT Gasparrini A Tong SL Molina T. Global, regional, and national burden of mortality associated with non-optimal ambient temperatures from 2000 to 2019: a three-stage modelling study. Lancet Planet Health. (2021) 5:E415–25. doi: 10.1016/S2542-5196(23)00143-2 34245712

[38] Gasparrini A Guo YM Hashizume M Lavigne E Zanobetti A Schwartz J. Mortality risk attributable to high and low ambient temperature: a multicountry observational study. Lancet. (2015) 386:369–75. doi: 10.1016/S0140-6736(14)62114-0 26003380 PMC4521077

[39] Bhaskaran K Gasparrini A Hajat S Smeeth L Armstrong B. Time series regression studies in environmental epidemiology. Int J Epidemiol. (2013) 42:1187–95. doi: 10.1093/ije/dyt092 23760528 PMC3780998

[40] Zhao DS Zhang XL Xie MY Cheng J Zhang H Wang S. Is greater temperature change within a day associated with increased emergency admissions for schizophrenia? Sci Total Environ. (2016) 566–567:1545–51. doi: 10.1016/j.scitotenv.2016.06.045 27320736

[41] Fan L Zhang DY. Study on the influence mechanism and spatial differentiation characteristics of Beijing’s neighborhood vitality – based on multi-scale geographically weighted regression. Urban Plan. (2022) 46:27–37. doi: 10.11819/cpr20220502a

[42] Fotheringham AS Yang WB Kang W. Multiscale geographically weighted regression (MGWR). Ann Am Assoc Geogr. (2017) 107:1247–65. doi: 10.1080/24694452.2017.1352480

[43] Shen TY Yu HC Zhou L Gu HY He HH. The influence mechanism of second-hand residential prices in Beijing – a study based on multi-scale geographically weighted regression model (MGWR). Econ Geogr. (2020) 40:75–83. doi: 10.15957/j.cnki.jjdl.2020.03.009

[44] Zhu XM Song XN Leng P Hu RH. Spatial downscaling of land surface temperature with the multi-scale geographically weighted regression. Natl Remote Sensing Bull. (2021) 25:1749–66. doi: 10.11834/jrs.20211202

[45] Phung D Hien TT Linh HN Luong LMT Morawska L Chu C. Air pollution and risk of respiratory and cardiovascular hospitalizations in the most populous city in Vietnam. Sci Total Environ. (2016) 557–558:322–30. doi: 10.1016/j.scitotenv.2016.03.070 27016680

[46] Luong LTM Dang TN Huong NTT Phung D Tran LK Dung DV. Particulate air pollution in Ho chi minh city and risk of hospital admission for acute lower respiratory infection (ALRI) among young children. Environ Pollut. (2020) 257:113424. doi: 10.1016/j.envpol.2019.113424 31672367

[47] Szyszkowicz M Kousha T Castner J Dales R. Air pollution and emergency department visits for respiratory diseases: a multi-city case crossover study. Environ Res. (2018) 163:263–9. doi: 10.1016/j.envres.2018.01.043 29459308

[48] Li X. Yan Q. P. Luo C. (2023), Impact of built environment on flow of transfer passengers between subway and bus considering spatial heterogeneity. J Transp Syst Eng Inf Technol. 23:100–10. doi: 10.16097/j.cnki.1009-6744.2023.02.011

[49] Jacob DJ Winner DA. Effect of climate change on air quality. Atmos Environ. (2009) 43:51–63. doi: 10.1016/j.atmosenv.2008.09.051

[50] Liu HY Jacob DJ Bey I Yantosca RM. Constraints from ^210^Pb and ^7^be on wet deposition and transport in a global three-dimensional chemical tracer model driven by assimilated meteorological fields. J Geophys Res Atmos. (2001) 106:12109–28. doi: 10.1029/2000JD900839

[51] Chen CQ Chen YQ Tang SJ Wu SJ. Analysis of effect of meteorological factor on air quality of Wuhan in 2001-2010. Environ Sci Technol. (2013) 36:130–3. doi: 10.3969/j.issn.1003-6504.2013.05.026

[52] Chen RJ Chu C Tan JG Cao JS Song WM Xu XH. Ambient air pollution and hospital admission in Shanghai. China J Hazardous Mater. (2010) 181:234–40. doi: 10.1016/j.jhazmat.2010.05.002 20537796

[53] Gu LJ Yu C. Analysis of the current situation of building energy consumption data and energy consumption statistics in China. Energy China. (2011) 2:38–41. doi: 10.3969/j.issn.1003-2355.2011.02.008

[54] Miao Z Baležentis T Shao S Chang D. Energy use, industrial soot and vehicle exhaust pollution—China’s regional air pollution recognition, performance decomposition and governance. Energy Econ. (2019) 83:501–14. doi: 10.1016/j.eneco.2019.07.002

[55] Shen F Jia YP Zhang Y Zhao Y Huang L Li RL. Indoor PM2.5 pollution levels and influencing factors in public places in Beijing in winter. J Environ Health. (2014) 3:262–3. doi: 10.16241/j.cnki.1001-5914.2014.03.011

[56] Liu ZX Wang GZ Liu LH. The analysis of pollution level of PM10 and PM2.5 in large and moderate scale shopping Centers. J Environ Health. (2006) 4:336–8. doi: 10.16241/j.cnki.1001-5914.2006.04.023

[57] Miller MR Newby DE. Air pollution and cardiovascular disease: car sick. Cardiovasc Res. (2020) 116:279–94. doi: 10.1093/cvr/cvz228 31583404

[58] Carozzi F Roth S. Dirty density: air quality and the density of American cities. J Environ Econ Manag. (2023) 118:102767. doi: 10.1016/j.jeem.2022.102767

[59] Borck R Schrauth P. Population density and urban air quality. Reg Sci Urban Econ. (2021) 86:103596. doi: 10.1016/j.regsciurbeco.2020.103596

[60] Wu D Gong JH Liang JM Sun J Zhang GY. Analyzing the influence of urban street greening and street buildings on summertime air pollution based on street view image data. ISPRS Int J Geo Inf. (2020) 9:500. doi: 10.3390/ijgi9090500

[61] Jim CY Chen WY. Assessing the ecosystem service of air pollutant removal by urban trees in Guangzhou (China). J Environ Manag. (2008) 88:665–76. doi: 10.1016/j.jenvman.2007.03.035 17499909

[62] Wang WJ Tian PL Zhang JH Agathokleous E Xiao L Koike T. Big data-based urban greenness in Chinese megalopolises and possible contribution to air quality control. Sci Total Environ. (2022) 824:153834. doi: 10.1016/j.scitotenv.2022.153834 35157858

[63] Sundell J Levin H Nazaroff WW Cain WS Fisk WJ Grimsrud DT. Ventilation rates and health: multidisciplinary review of the scientific literature. Indoor Air. (2011) 21:191–204. doi: 10.1111/j.1600-0668.2010.00703.x 21204989

