# Supplementary material for: Research on the cascading mechanism of “urban built environment-air pollution-respiratory diseases”: a case of Wuhan city
Source: Front Public Health. 2024 Mar 22;12:1333077. doi: 10.3389/fpubh.2024.1333077 (PMC10995312; doi:10.3389/fpubh.2024.1333077)
Supplement: Supplementary file 1 [file Data_Sheet_1.ZIP › Figure S2.docx]

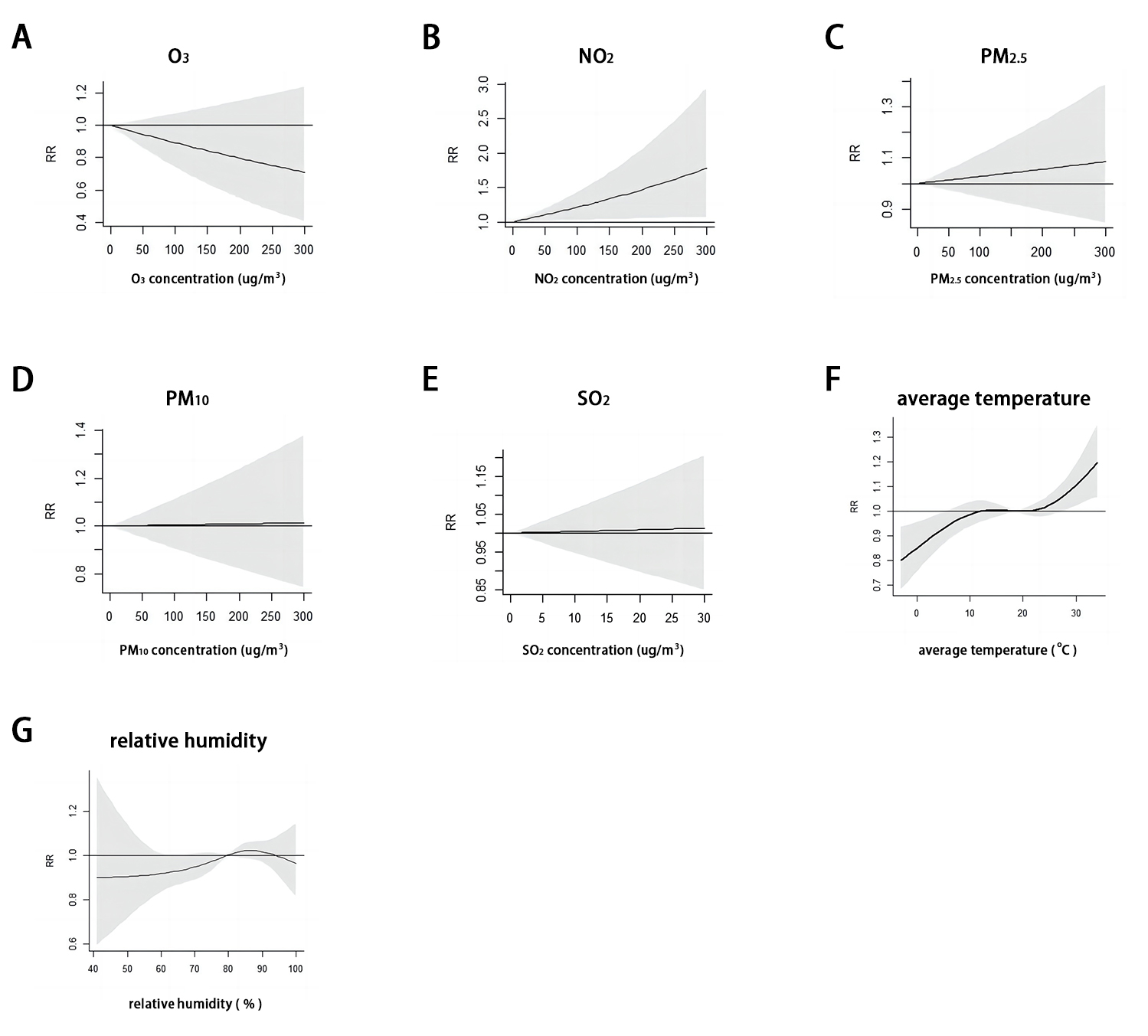


Figure 3

2D plots of effect of air pollution on the number of respiratory disease hospitalizations (A) the impact of O_3_ on hospitalization for respiratory diseases, (B) the impact of NO_2_ on hospitalization for respiratory diseases, (C) the impact of PM_2.5_ on hospitalization for respiratory diseases, (D) the impact of PM_10_ on hospitalization for respiratory diseases, (E) the impact of SO_2_ on hospitalization for respiratory diseases, (F) the impact of average temperature on hospitalization for respiratory diseases, (G) the impact of relative humidity on hospitalization for respiratory diseases.

SO_2_ indicates sulfur dioxide, NO_2_ indicates nitrogen dioxide, PM_10_ and PM_2.5_ indicates particulate matter, O_3_ indicates ozone, RH indicates relative humidity, and RR indicates relative risk.
