# Supplementary figures and images for: Research on the cascading mechanism of “urban built environment-air pollution-respiratory diseases”: a case of Wuhan city
Source: Front Public Health. 2024 Mar 22;12:1333077. doi: 10.3389/fpubh.2024.1333077 (PMC10995312; doi:10.3389/fpubh.2024.1333077)

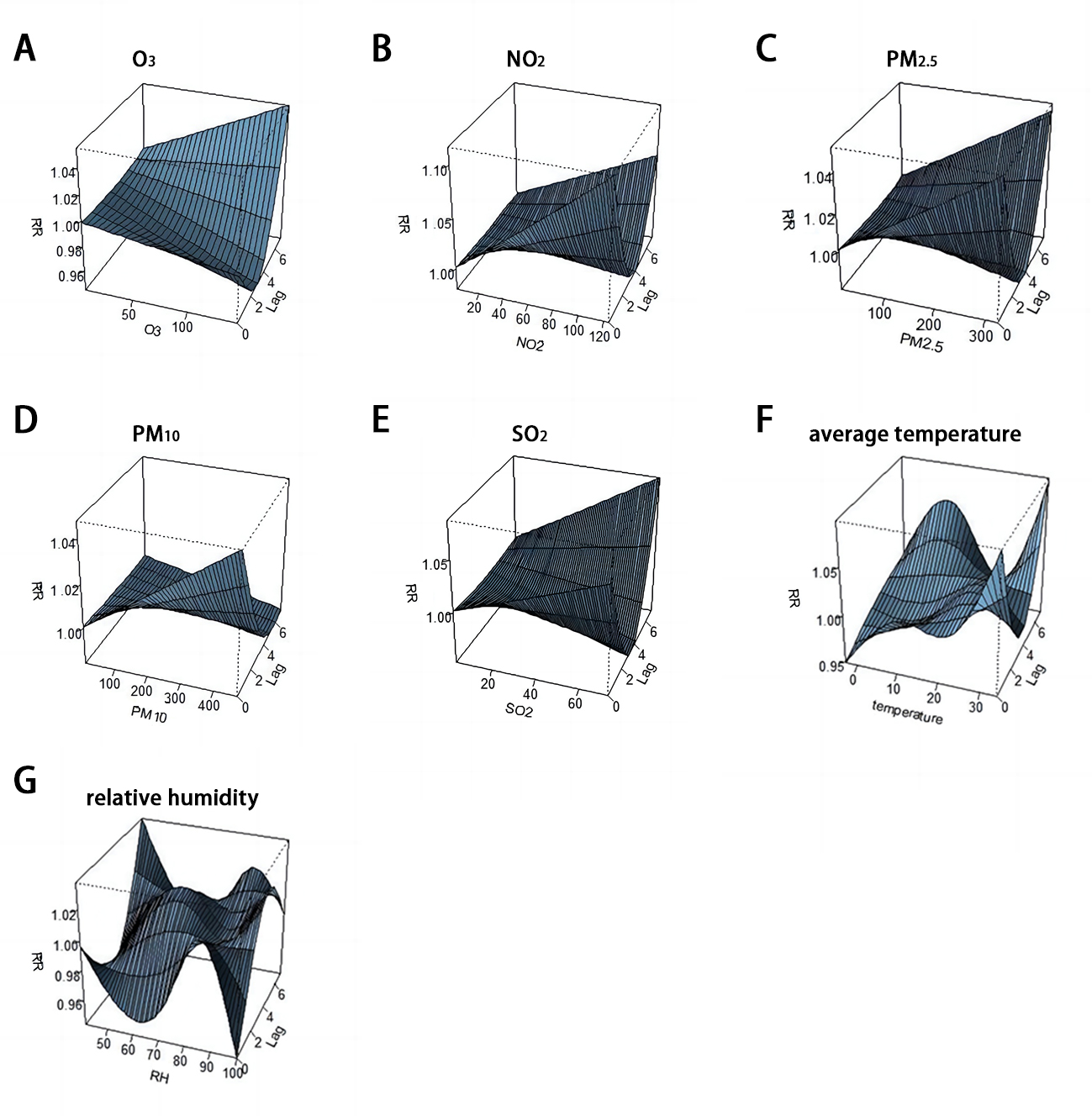

Supplement: Supplementary file 1 [file Data_Sheet_1.ZIP › Figure S1.jpg]

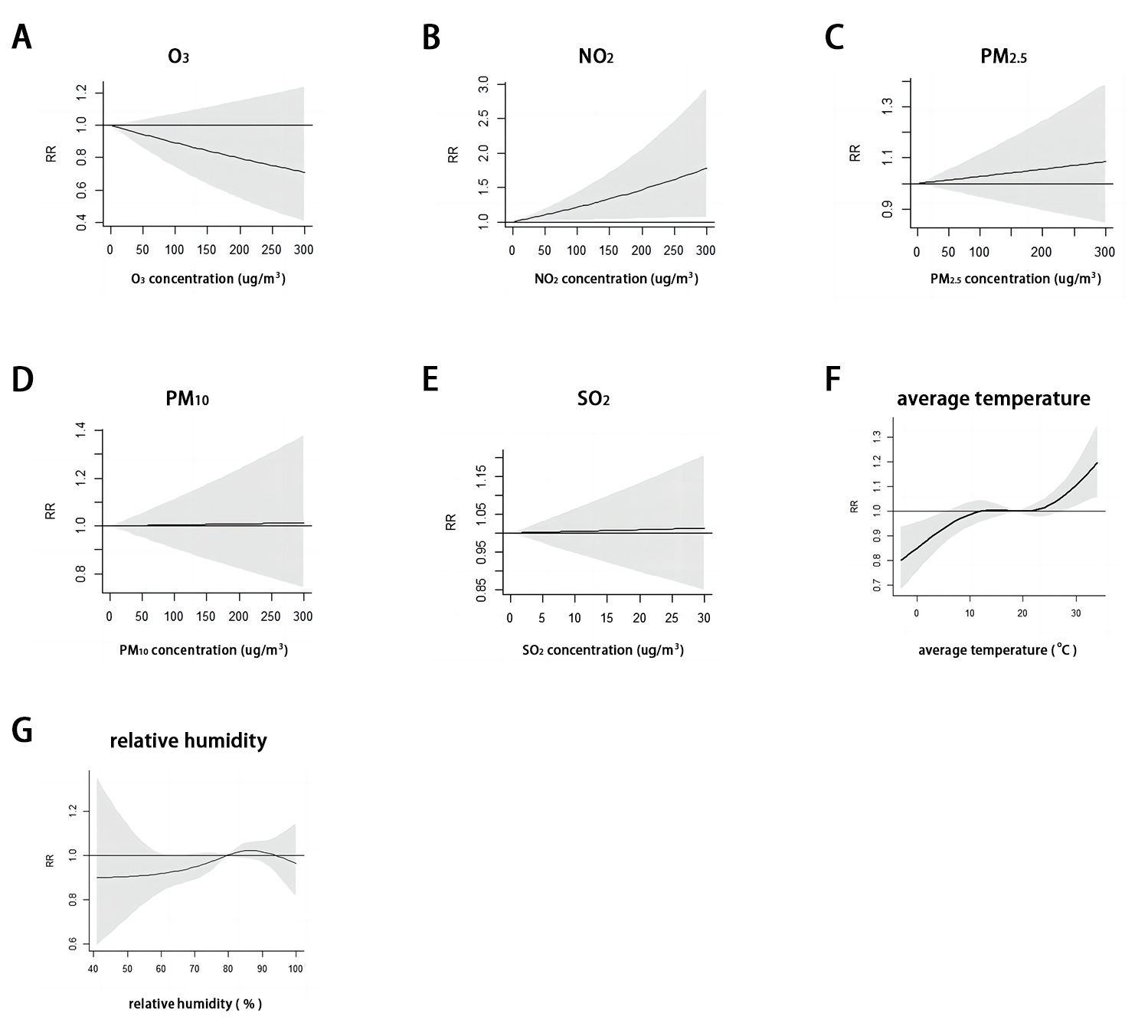

Supplement: Supplementary file 1 [file Data_Sheet_1.ZIP › Figure S2.jpg]
